# A Comprehensive View of the Epigenetic Landscape Part I: DNA Methylation, Passive and Active DNA Demethylation Pathways and Histone Variants

**DOI:** 10.1007/s12640-014-9497-5

**Published:** 2014-11-02

**Authors:** Anna Sadakierska-Chudy, Richard M. Kostrzewa, Małgorzata Filip

**Affiliations:** 1 Laboratory of Drug Addiction Pharmacology, Institute of Pharmacology Polish Academy of Sciences, Smetna Street 12, 31-343 Kraków, Poland; 2 Department of Biomedical Sciences, Quillen College of Medicine, East Tennessee State University, Johnson City, TN 37614 USA; 3 Department of Toxicology, Faculty of Pharmacy, Jagiellonian University, Medical College, Medyczna 9, 30-688 Kraków, Poland

**Keywords:** Cytosine variants, DNA methylation, DNA methyltransferases, Histone variants, Passive and active demethylation, TET family enzymes

## Abstract

In multicellular organisms, all the cells are genetically identical but turn genes on or off at the right time to promote differentiation into specific cell types. The regulation of higher-order chromatin structure is essential for genome-wide reprogramming and for tissue-specific patterns of gene expression. The complexity of the genome is regulated by epigenetic mechanisms, which act at the level of DNA, histones, and nucleosomes. Epigenetic machinery is involved in many biological processes, including genomic imprinting, X-chromosome inactivation, heterochromatin formation, and transcriptional regulation, as well as DNA damage repair. In this review, we summarize the recent understanding of DNA methylation, cytosine derivatives, active and passive demethylation pathways as well as histone variants. DNA methylation is one of the well-characterized epigenetic signaling tools. Cytosine methylation of promoter regions usually represses transcription but methylation in the gene body may have a positive correlation with gene expression. The attachment of a methyl group to cytosine residue in the DNA sequence is catalyzed by enzymes of the DNA methyltransferase family. Recent studies have shown that the Ten-Eleven translocation family enzymes are involved in stepwise oxidation of 5-methylcytosine, creating new cytosine derivatives including 5-hydroxymethylcytosine, 5-formylcytosine, and 5-carboxylcytosine. Additionally, histone variants into nucleosomes create another strategy to regulate the structure and function of chromatin. The replacement of canonical histones with specialized histone variants regulates accessibility of DNA, and thus may affect multiple biological processes, such as replication, transcription, DNA repair, and play a role in various disorders such as cancer.

## Introduction

In the last decade, epigenetics has become an important topic of genetic research. The classical definition of epigenetics refers to the mitotically and/or meiotically heritable changes in gene activity that does not involve alterations in DNA sequence. This definition emphasizes the heritability of the cellular phenotype, and therefore, it only includes changes in the germ line that can be passed down from generation to generation and changes in dividing cells that can be transferred to daughter cells. Currently, we know that epigenetic changes can be induced by environmental factors at different times in life and are potentially reversible. In 2007, Brenda Weis proposed the broader term of epigenetics that refers to “the study of regulation of gene activity that is not dependent on gene sequence and includes heritable and non-heritable alterations in gene activity and transcriptional potential of a cell” (Brenda Weis at the “Diet, Epigenetic Events, and Cancer Prevention Symposium” on September 27th, 2007, in Washington, D.C./http://prevention.cancer.gov/files/news-events/100908_epigenetics%20meeting%20report%20Sept%202007.pdf).

Epigenetic control operates on three major levels, i.e., on DNA, histones, and nucleosomes. The relationships among these various epigenetic elements are currently being extensively investigated. In this review, data from the literature are analyzed to discuss the significance of DNA methylation and demethylation, cytosine derivatives as well as histone variants in the epigenetic regulation of the genome.

## DNA Level

### DNA Methylation

DNA methylation is a biochemical process crucial for normal development in higher organisms, and it is the most thoroughly studied epigenetic mark. Methylation entails the covalent attachment of a methyl (CH_3_) group to the C5 position of a cytosine residue, forming 5-methylcytosine (5mC).
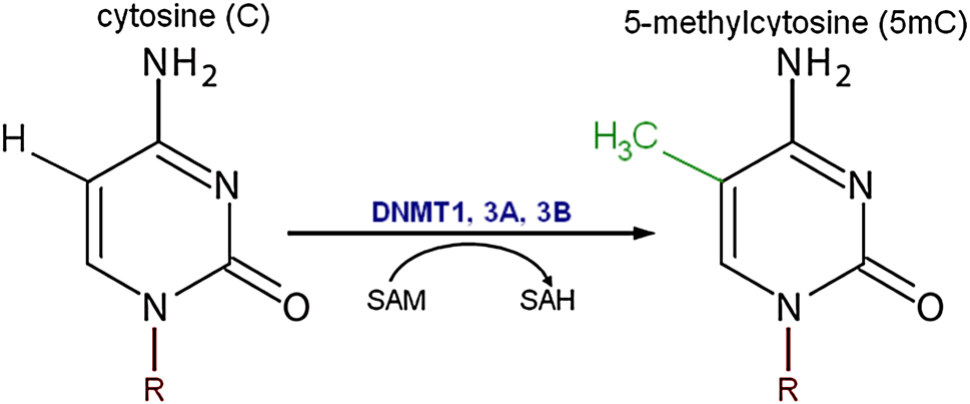


In some organisms, this modification is so frequent that it is denoted as the fifth nucleotide. The methyl group is transferred from *S*-adenosyl-l-methionine (SAM) to cytosine by the DNA methyltransferase (DNMT) family of enzymes: DNMT1, DNMT2, DNMT3A, DNMT3B, and DNMT3L (Jin et al. 2011). DNMT1 preferentially methylates hemimethylated cytosines in CpG dinucleotide sequences, maintaining the methylation pattern during replication (Probst et al. 2009). In contrast to DNMT1, DNMT3A and 3B prefer unmethylated CpG dinucleotides and perform de novo methylation in early development (Li 2002). Thus, DNMT1 acts primarily as a maintenance methyltransferase during DNA synthesis, and DNMT3A and DNMT3B act as de novo enzymes in development. A growing body of evidence suggests that DNMT1 may also be necessary for de novo methylation of genomic DNA (Egger et al. 2006) and that DNMT3A and DNMT3B are also responsible for the maintenance of methylation during cell replication (Riggs and Xiong 2004). It is worth noting that DNMT2 displays weak DNA methyltransferase activity but actually functions as an RNA methyltransferase. The DNMT2 enzyme specifically methylates cytosine-38 in the anticodon loop of aspartic acid transfer RNA that protects tRNAs from cleavage under stress conditions (Goll et al. 2006; Schaefer et al. 2010).

**Fig. 1:**
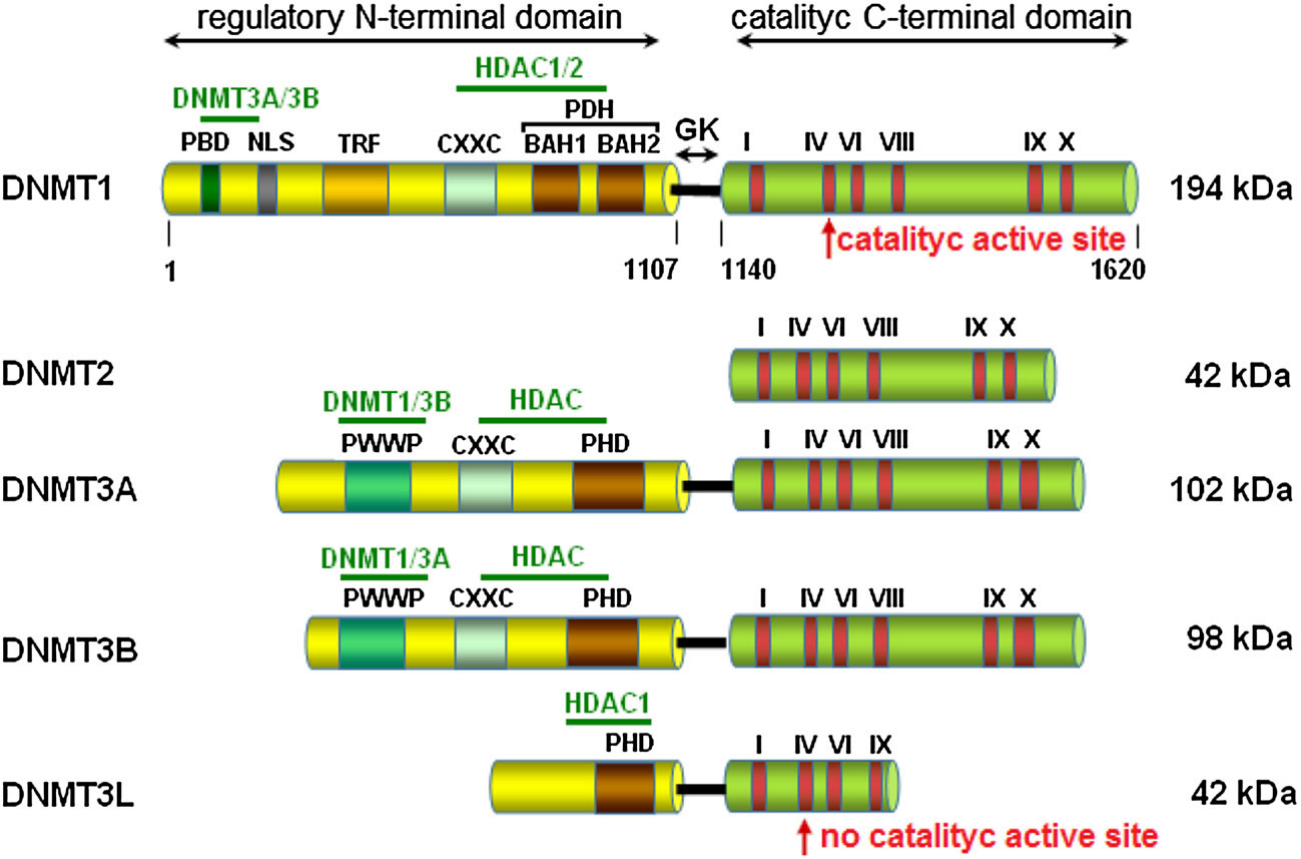
Schematic structure of mammalian DNMT family members. DNMT1, the first described methyltransferase, preferentially methylates hemimethylated DNA (Robertson 2001). DNMT2 lacks the N-terminal domain, while C-terminal domain contains the full set of sequence motifs but has not been shown to have transmethylase activity (Bestor 2000). DNMT3A and DNMT3B have similar domain arrangements and an equal preference for hemimethylated and unmethylated DNA (Robertson 2001). DNMT3L, being closely related to the catalytic domain of DNMT3A/3B, lacks canonical DNA cytosine−methyltransferase motifs (Bestor 2000). Its N-terminal regulatory domains exhibit little similarity but the catalytic domains of DNMTs are conserved. The N-terminal domain possesses: PBD—proliferating cell nuclear antigen (PCNA) binding domain, NLS—nuclear localization signal, TRF—targeting replication foci, CXXC—cysteine rich, zinc finger DNA-binding motif, PBH—polybromo homology domain, PWWP—tetrapeptide domain containing proline−tryptophan−tryptophan−proline motif. The N-terminal and C-terminal domains are linked by dinucleotide repeats: GK—glycine−lysine repeat. The C-terminal domain consists of six most conserved amino acid motifs (motif I and X form SAM binding site, motif IV binds cytosine at the active site). Mapped interaction sites of DNMTs and HDACs (histone deacetylases) are indicated in the above diagrams. The borders of the DNMT1 domains are marked according Song et al. (2011)

A recent finding has suggested that DNMT2 might be involved in the mammalian paramutation pathway, by protecting small RNA molecules against endonucleolytic cleavage (Adams and Meehan 2013; Kiani et al. 2013), and thus it might induce heritable epigenetic phenotypes. DNMT3L, although it shares homology with DNMT3A and 3B, has no catalytic activity. Instead, DNMT3L increases the ability of DNMT3A and B to bind to methyl groups, thus facilitating methylation in vivo (Bird 2002; Jin et al. 2011). Moreover, DNMT3L recognizes nucleosomes with an unmethylated histone H3 lysine 4 (H3K4) and recruits DNMT3A and DNMT3B to their targets (Saitou et al. 2012). Structural and functional domains of mammalian DNMTs are shown in Fig. 1.

The level of 5mC affects gene expression, and deregulation of cytosine methylation may play a role in development, cellular differentiation, or disease (Santos-Rebouc and Pimentel 2007; Aguilera et al. 2010; Hackett and Surani 2013). The DNA methylation level can affect transcriptional activities, hypermethylation (a surplus of methyl groups) of promoter regions, and is generally associated with transcriptional silencing, but hypomethylation (a deficit of methyl groups) causes an increased level of gene expression (Crider et al. 2012). Approximately, 2–8 % of the cytosines in the mammalian genome are methylated, mostly in CpG sequences (Zhu 2009; Varriale 2014). In the human genome, CpG dinucleotides are distributed asymmetrically among GC-rich and -poor DNA regions, and not all CpG sites are methylated. The pattern of DNA methylation varies in different cell types and is tissue specific. For example, in differentiated mammalian cells, cytosine residues in GC-rich regions (which typically contain more than 50 % GC) are usually methylated. DNA regions that contain a high frequency of CpG sites are so-called CpG islands (CGIs) and represent an important feature of the mammalian genome. They are located in promoters, preferentially near the transcription start sites (TSSs) of >50 % of human genes. CGI methylation is lower at promoters and higher in gene bodies and intergenic regions. CGI-rich promoters are largely free of DNA methylation due to the abundance of GC-rich transcription factor-binding sites (Deaton and Bird 2011). Methylation of DNA cytosine residues at promoter regions usually correlates with a higher order of chromatin state and repression mRNA transcription. However, Niesen et al. (2005) revealed that a sequence-specific DNA-binding protein can facilitate transcriptional activation of methylated promoter. Interestingly, recent findings suggest that in undifferentiated stem cells, cytosines outside of CpG sites can be methylated as well, and this process is particularly important for the proper regulation of gene expression in embryonic stem cells (ESCs) (Lister et al. 2009). As previously mentioned, gene bodies are highly methylated but the role of methylation remains largely unresolved. Some studies have begun to decipher molecular implications of gene body methylation. For example, methylation in the gene body contributes to the suppression of transcriptional noise (Huh et al. 2011) and might stimulate transcription elongation (Jones 2012). A recent study has suggested that exons are methylated at higher levels than introns and possibly play a role in the regulation of mRNA splicing (Laurent et al. 2010). More details about genomic locations of DNA methylation and its consequence can be found in excellent recent reviews (Estécio and Issa 2011; Moore et al. 2013).

DNA methylation has been considered a stable, persistent and heritable mark; therefore, methyl groups are added but not removed. Recent data have indicated that transcription factors and related proteins not only protect sequences from methylation but also initiate active DNA demethylation (Stadler et al. 2011). Both passive demethylation during replication and active demethylation take place in eukaryotic cells. For example, DNA methylation patterns undergo reprogramming during the establishment of primordial germ cells (PGCs) as well as after fertilization (Branco et al. 2011; Saitou et al. 2012). Surprisingly, the establishment of DNA methylation patterns occurs during development and differentiation of the central nervous system, where it has been implicated in synaptic plasticity, learning, and memory. In the human brain, DNA methylation changes are strongly correlated with age (Hernandez et al. 2011). In turn, pathological activation of DNMTs and aberrant 5mC formation may cause neurodegradation and apoptotic neuronal death (Chestnut et al. 2011; Hernandez and Singleton 2012).

DNA methylation influences gene expression not only by impeding the binding of specific transcription factors but also by recruiting chromatin-modifying proteins. DNA methylation also determines the histone modification patterns and the DNMTs and methyl-CpG-binding domain (MBD) proteins that help to recruit repressor complexes containing histone deacetylases (HDACs) (Fuks et al. 2003). Conversely, interactions between DNMT1, G9a (methyltransferase H3K9), and the replication complex lead to dimethylation of histone H3 lysine 9 (H3K9me2), a repressive epigenetic mark. Methylated H3K9 is bound by heterochromatin protein 1 (HP1), which interacts directly with DNMT1, resulting in cytosine methylation (Smallwood et al. 2007; Saitou et al. 2012). The interaction of the H3K9 methyltransferases (SUV39H1 and ESET) with DNMT3A and DNMT3B can also cause DNA methylation at H3K9me2 (Fuks et al. 2003). Notably, chromatin organization differs between CpG and non-CpG promoters. GC-rich DNA is preferentially bound by CXXC domain proteins that can recruit chromatin modifiers, including the CXXC finger protein 1 (Cfp1) subunit of the H3K3me3 methyltransferase complex and KDM2A, an H3K36me2 demethylase (Vavouri and Lehner 2012). In addition to participating in the histone modifications, DNA methylation may influence the incorporation of histone variant H2A.Z into nucleosomes. A growing body of evidence suggests that the H2A.Z is excluded from methylated DNA and the global anticorrelation between DNA methylation and H2A.Z is observed (Conerly et al. 2010; Weber and Henikoff 2014).

Taken together, DNA methylation affects the interaction between the histone and DNA, resulting in either activation or repression of transcription. It is well known that the disruption of methylation patterns can cause many diseases including cancer, autoimmune disease, as well as chromosomal instability, and mental retardation syndromes (Dobrovic 2010; Javierre et al. 2011). In humans, mutations in genes, including DNMTs and methyl-CpG binding proteins (MBPs), could have profound impact on specific DNA methylation patterns leading to epigenetic diseases (Santos-Rebouc and Pimentel 2007). Up to now, more studies have signified that life style and environmental factors, such as nutrient supply, drugs, pollutants, pathogens, sex hormones, radiation, heavy metals, and early stress can modulate DNA methylation (Javierre et al. 2011; Lim and Song 2012). Interestingly, certain dietary constituents (e.g., folate and bioactive components) may alter genomic and gene-specific DNA methylation levels during embryonic development and adult life (Aguilera et al. 2010; Choi and Friso 2010; McKay and Mathers 2011). Concerning the reversible nature of DNA methylation, it seems to be attractive for epigenetic modulation (Egger et al. 2004; Yang et al. 2010).

### Cytosine Variants

It has long been known that cytosine can exist in one of two functional states, unmethylated or methylated. Moreover, mechanisms of DNA methylation are among the best understood epigenetic phenomena. Recently, several cytosine variants, including 5-hydroxymethylcytosine (5hmC), 5-formylcytosine (5fC), 5-carboxylcytosine (5caC), and 3-methylcytosine (3mC), were identified.
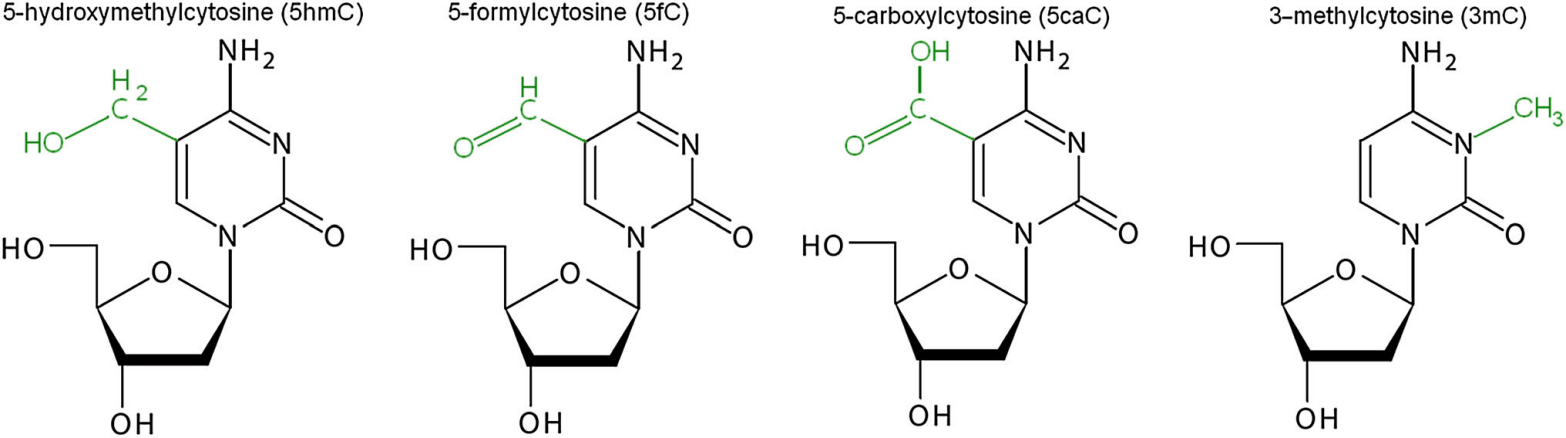


#### 5-Hydroxymethylcytosine (5hmC)

**Fig. 2:**
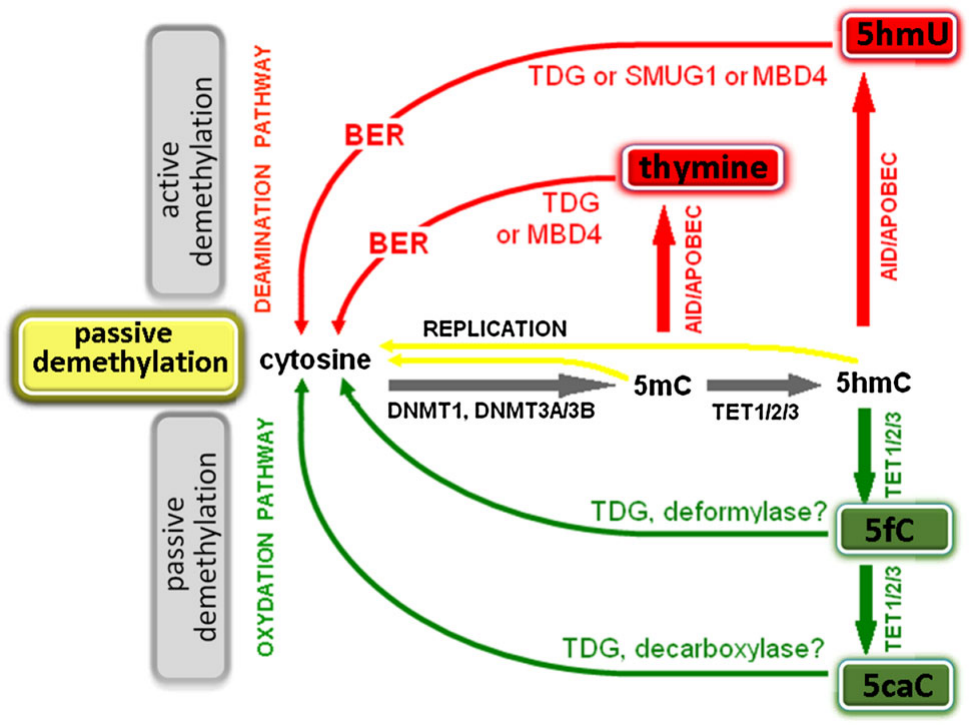
Passive and active DNA demethylation pathways. Passive DNA demethylation is caused by a reduction in activity or absence of DNMTs (*yellow arrows*). Active demethylation via oxidation pathway (*green arrows*): TET enzymes can hydroxylate methylcytosine (5mC) to form 5-hydroxymethylcytosine (5hmC); further oxidation produces 5-formylcytosine (5fC) and 5-carboxylcytosine (5caC). 5fC and 5caC can be actively removed by the DNA glycosylases. In addition, a putative deformylase may convert 5fC to C and decarboxylase convert 5caC to C. Active demethylation via deamination pathway (red arrows): AID/APOBEC family members can deaminate 5mC or 5hmC to form thymidine or 5-hydroxymethyluracil (5hmU). These intermediates are replaced by cytosine during base excision repair (BER) mediated by the uracil-DNA glycosylase (UDG) family, like TDG or SMUG1 as well as MBD4 (specifically recognize thymine and 5hmU). AID activation-induced deaminase, APOBEC apolipoprotein B mRNA-editing enzyme complex, BER—base excision repair, DNMT1/3A/3B—DNA methyltransferase, MBD4—methyl-binding domain protein 4, SMUG1—single-strand specific monofunctional uracil-DNA glycosylase, TET1/2/3—ten-eleven methylcytosine dioxygenase family, TDG—thymine-DNA glycosylase (Color figure online)

5-Hydroxymethylcytosine was discovered 60 years ago in T2 bacteriophage (Wyatt and Cohen 1952), and 20 years later Penn et al. found 5hmC base in mammalian cells (Penn et al. 1972). These early findings could not be replicated in later studies until 2009, when two independent groups showed that 5hmC exists in mouse Purkinje neurons (Kriaucionis and Heintz 2009) and in ESCs (Tahiliani et al. 2009). Currently, 5hmC is regarded as the “sixth” base of the genome of higher organisms (Münzel et al. 2010). The levels of 5hmC in the genome are relatively low and account for ~0.4 % of all cytosines compared to the ~10 % that are 5mC (Branco et al. 2011). 5hmC constitutes approximately 40 % of the modified cytosines in mouse brain, and the amount increases during maturation in both the hippocampus and the cerebellum (Szulwach et al. 2011). Recently, it has been confirmed that 5hmC is generated by the Ten-Eleven Translocation (TET) enzymes that are Fe(II) and α-oxoglutarate-dependent dioxygenases. The TET subfamily, including TET1, TET2, and TET3, catalyzes the conversion of 5mC–5hm in vitro and in vivo (Ito et al. 2010; Branco et al. 2011) and may be engaged in the further oxidation of 5hmC–5fC and 5caC (He et al. 2011; Ito 2011) (Fig. 2). The TET proteins contain iron and oxyglutarate domains as well cysteine-rich regions that are most likely involved in DNA binding (Iyer et al. 2009). Moreover, TET1 and TET3 contain CXXC zinc finger domains, which allow binding to unmethylated, methylated and hydroxymethylated DNA.

Other CXXC-containing proteins, for example DNMT1, almost solely bind to unmethylated DNA; therefore, poor recognition of 5hmC could lead to passive demethylation (Valinluck and Sowers 2007). The level of 5hmC in adult tissues is between 0.03 and 0.69 % with the highest levels (0.4–0.7 %) in the central nervous system (Globisch et al. 2010). The biological role of 5hmC is still unclear. It has been postulated that 5hmC could be an intermediate in active DNA demethylation, and it may play an important role in gene regulation (Tahiliani et al. 2009; Wu and Zhang 2010). It has been observed that 5hmC is enriched in the body of the active genes and at the TSSs of transcriptionally inactive genes (Song et al. 2010; Wu et al. 2011a). *In vitro* analysis revealed that 5hmC in the gene body prevents the binding of MBD proteins, which act as transcriptional repressors (Valinluck et al. 2004; Jin et al. 2010). The level of 5hmC in the gene body might modify the accessibility of chromatin to the transcriptional machinery. Nestor et al. have demonstrated that 5hmC patterns are tissue specific. The global content of 5hmC varies markedly between tissues and does not correlate with global 5mC levels (Nestor et al. 2012). Chen et al. (2012) have demonstrated that aging increases both global- and locus-specific 5hmC content in the mouse hippocampus.

It is possible that 5hmC initiates the pathway of passive or active DNA demethylation by excluding DNMT1 and the MBD proteins from methylating cytosine, and it may recruit other unknown 5hmC-specific effector proteins (Stroud et al. 2011). Recent in vitro studies have revealed that TET proteins could contribute to the removal of methylated cytosine (He et al. 2011; Ito et al. 2011; Matarese et al. 2011). This enzyme family has the capacity to oxidize 5mC not only to 5hmC but also to 5-formylcytosine and 5-carboxylcytosine. Other researchers have shown that thymine-DNA glycosylase (TDG) belonging to the uracil-DNA glycosylase (UDG) superfamily can recognize and excise 5fC and 5caC; thus, the base excision repair (BER) system could be a trigger (Ooi and Bestor 2008; He et al. 2011; Matarese et al. 2011). The crystal structure of human TDG revealed a binding pocket that can accommodate 5caC which facilitates its cleavage (Zhang et al. 2012; Kohli and Zhang 2013). Furthermore, TDG can remove T:G or hmU:G mismatches generated by enzymatic deamination of 5mC to thymine and 5hmC to 5-hydroxymethyluracil (5hmU) (Shen et al. 2014). In addition, alternative UDG glycosylases including methyl-CpG-binding domain protein 4 (MBD4) and single-strand-selective monofunctional uracil-DNA glycosylase 1 (SMUG1) can be involved in active DNA demethylation pathway (Shen et al. 2014). Recent studies have reported that the hydroxylation of 5mC mediated by the Tet1 protein promotes active DNA demethylation in the adult brain by deaminating cytosine residue to uracil by the activation-induced deaminase (AID)/apolipoprotein B mRNA-editing enzyme complex (APOBEC) family, and then deaminated cytosine residue is excised by DNA glycosylases and repaired by the BER pathway (Guo et al. 2011). Potential mechanisms responsible for passive and active demethylation are presented in Fig. 2.

#### 5-Formylcytosine (5fC)

5-Formylcytosine is one of the DNA base variants produced by oxidation of 5hmc by the TET family of enzymes (Ito et al. 2011). Thin layer chromatography and tandem liquid chromatography−mass spectrometry has revealed 5fC in mouse ESCs and in brain cortex (Raiber et al. 2012). The levels of 5fC are estimated to be from 0.02 to 0.002 % of the genomic DNA of ES cells and are 10- to 100-fold lower than the levels of 5hmC (Ito et al. 2011; Pfaffeneder et al. 2011). These levels seem reasonable because TET1 and TET2 are highly expressed and most likely play roles in DNA methylation reprogramming and cell differentiation (Koh et al. 2011). Indeed, during differentiation, levels of 5fC decrease, suggesting its participation in development and germ cell programming (Pfaffeneder et al. 2011). A recent study has reported that CGI promoters were more enriched in 5fC levels than in 5hmC or 5mC levels, which correlated with active gene expression. Moreover, TDG was shown to be actively involved in the removal of 5fC marks in CGIs, exons, and promoter regions (Raiber et al. 2012). Therefore, 5fC excision may help to establish correct methylation patterns during cell-specific developmental programs. Surprisingly, 5fC-enriched promoter regions overlap with H3K4me3, suggesting cross-talk between these marks in transcriptionally active genes.

#### 5-Carboxylcytosine (5caC)

5-Carboxylcytosine is one of the intermediates in active DNA demethylation and is produced by TET-mediated enzymatic oxidation from 5fC. The TET3 protein is most likely responsible for this conversion (Gu et al. 2011). To date, 5caC has been found in ESCs and in mouse pre-implantation embryos (Inoue et al. 2011; He et al. 2011). Alioui and co-workers have shown that 5caC is detectable in the somatic cells of amphibian ovaries and is primarily localized to gene-rich euchromatic regions similar to 5hmC (Alioui et al. 2012). This study also demonstrated that TDG glycosylase can initiate the BER pathway and cleave 5caC both in vitro and in vivo, but the MBD4 enzyme exhibited no activity toward 5caC. Interestingly, 5caC levels increased when TDG was depleted in mouse ES cells; thus, TDG is most likely not the only enzyme capable of processing 5caC (He et al. 2011). It is not known whether TDG is able to recognize and excise 5caC from duplex DNA and whether additional enzymes might be engaged in the conversion of 5caC in mammalian cells.

#### 3-Methylcytosine (3mC)

3-Methylcytosine is a DNA adduct created by spontaneous exposure to endogenous or environmental alkylating agents, leading to cytotoxicity and carcinogenesis. This mutagenic lesion can be directly repaired with the participation of the ABH3 or ABH2 DNA dioxygenases through the BER pathway in humans, or it can be dealkylated by AlkB in bacteria (Koivisto et al. 2004; Yi et al. 2012). Biochemical experiments indicate that ALKBH2 prefers double-stranded DNA (dsDNA) substrates, while ALKBH3 prefers single-stranded DNA (ssDNA) substrates, which are generated by the activating signal cointegrator complex (ASCC) (Dango et al. 2011; Yi et al. 2012). Dango et al. (2011) demonstrated that loss of ALKBH3 or ASCC3 significantly reduced cell proliferation in vitro and in vivo in xenograft models. Concurrently, the accumulation of endogenous 3meC in genomic DNA was observed. Additionally, ALKBH2 has been shown to play an efficient role in pediatric brain tumors during chemotherapy treatment, and the combination of an ALKBH2 knockdown and cisplatin chemotherapy seems to improve the efficacy of treatment (Cetica et al. 2009; Wu et al. 2011b). Taken together, these findings indicate an important role for alkylation repair in removing environmentally induced DNA lesions as well as in maintaining genome integrity and stability.

## Histone Variants

**Fig. 3:**
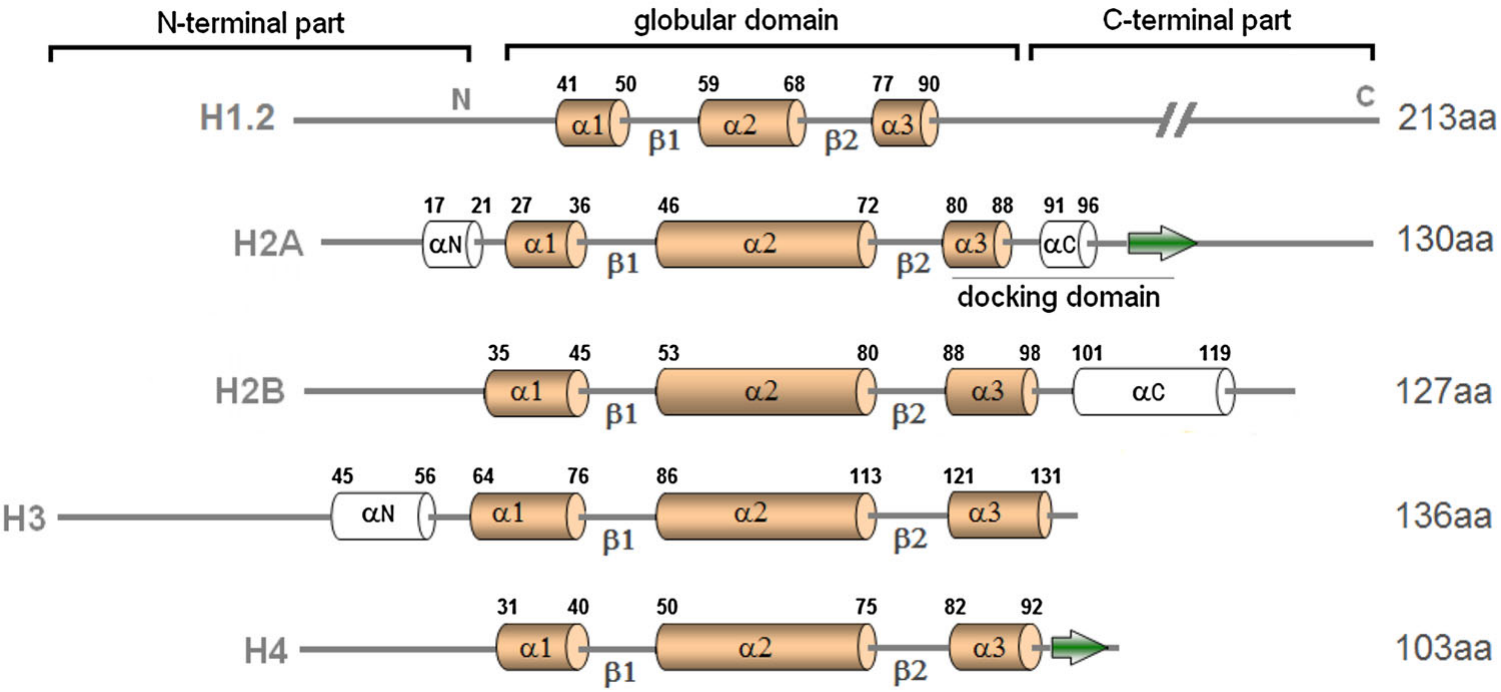
Schematic structure of the five histone proteins. The N-terminal part is flexible and positively charged and protrudes from the nucleosome. Two short helices, α-1 and α-2 have a length of 10–14 amino acid residues; central α-2 helix comprises ~28 amino acid residues (Luger 2001). The H2A-docking domain spans amino acids 82–119 and is implicated in both structural and functional properties of the nucleosome (Shukla et al. 2011). It stabilizes the wrapping of one helical turn of DNA around the histone octamer (Shukla et al. 2011) and the binding of H2A–H2B dimers to (H3–H4)_2_ tetramers (Bönisch and Hake 2012). In addition, the H2A C-terminus has also been found to be crucial for binding of the linker histone H1 to nucleosome (Vogler et al. 2010). α helices and β strands of the histone fold extensions are shown as *open boxes* and *arrows*, respectively

Histones are small, basic, and highly conserved proteins that serve as structural scaffolds for DNA packaging (Cooper 2000). A DNA molecule (~147 bp in length) wrapped around the octamer of a histone (two dimers of H2A–H2B and a heterotetramer (H3–H4)_2_) constitutes a nucleosome, the fundamental repeating unit of eukaryotic chromatin (Cooper 2000). Histone H1 binds to linker DNA (~50 bp) between nucleosomes, forming a macromolecular structure to help in further compaction of genomic DNA (Sancho et al. 2008). Histone proteins have a tripartite structure consisting of a central globular domain flanked by N- and C-terminal parts (Fig. 3). The unstructured tail located at the N-terminal portion protrudes away from the nucleosome and, therefore, is prone to a variety of post-translational modifications (PTMs) (Kouzarides 2007). The highly conserved globular domain, termed a helix-turn-helix, contains three α–helices separated by loop regions and is involved in histone–histone and histone−DNA interactions (Luger 2001). The C-terminal domains of all histones except histone H1 and H2A are relatively short (Vogler et al. 2010).

Histone tails have many positively charged amino acids (especially lysine and arginine), which facilitate their binding to the negatively charged DNA molecule and intranucleosomal interaction (Hansen 2002). The N-terminal histone tails have been studied extensively, but little is known about the function of the C-terminal part. Vogler et al. have shown that the H2A C-terminal tail plays a pivotal role in regulating chromatin structure and dynamics (Vogler et al. 2010). These experiments revealed that the H2A C-terminus is required for efficient nucleosome translocation by chromatin remodelers and acts as a novel recognition module for linker histone H1 (Vogler et al. 2010). It appears that the H2A C-terminal tail has a dual function. On the one hand, it provides stabilization of the nucleosomal core particle, and on the other hand, it participates in interactions with proteins that control chromatin dynamics and conformation.

There are highly similar forms of histones termed ‘histone variants’. It has been estimated that approximately 937 different variants of linker and core histones exist in various species. In humans, 57 histone variants are encoded by 94 genes, the majority of them being present in four clusters: cluster 1 on chromosome 6 (6p22), cluster 2 on chromosome 1 (1p21), cluster 3 on chromosome 1 (1q42), and cluster 4 on chromosome 12 (12p12–13). The incorporation of specific histone variants into nucleosomes has significant impacts on gene expression, heterochromatization, and the formation of specialized regions of the chromatin (Kamakaka and Biggins 2005; Pusarla and Bhargava 2005). The histone variants have recently emerged as important factors in regulating chromatin states and also DNA repair in response to genotoxic treatments (Malik and Henikoff 2003). Moreover, it is likely that histone variants, as potential drivers of cancer initiation and/or progression, thus may be utilized as prognostic indicators of cancer (Vardabasso et al. 2014).

### Histone H1

Histone H1 proteins consist of 194–346 amino acid residues, depending on the variant. Approximately 126 different members of the H1 family have been reported from diverse species thus far (http://www.actrec.gov.in/histome/). Eleven variants of histone H1 have been described in humans; these are coded by a single gene that exhibits either replication-dependent or replication-independent expression (Table 1). Three of the variants are testis-specific (i.e., HIST1H1T, H1FNT, and HILS1), one of them is oocyte-specific (H1foo), and the others are somatic variants. Linker histone H1 is involved in chromatin compaction and plays a role in the formation of higher-order chromatin structures (Millán-Ariño et al. 2014). The specific role of histone H1 variants is still far from clear, and genomic distribution of H1 is challenging due to the lack of variant-specific antibodies (Izzo et al. 2013).

**Table 1:** Variants of histone H1 in humans

Variants	Name of genes	Genomic location (Ensembl)	Protein length (aa) (Swiss-Prot)	Function (reference)
H1.0^b^	H1F0	22q13.1	194	Nucleosome spacing and chromatin compaction
H1.1^a^	HIST1H1A	6p22.2	215	Linker histones inhibit sliding of histone octamers, and it is postulated that they can inhibit chromatin remodeling (Clausell et al. 2009)
H1.2^a^	HIST1H1C	6p22.2	213	
H1.3^a^	HIST1H1D	6p22.2	221	However, a recent study suggested that H1.2–H1.5 are depleted from active promoters and gene regulatory elements but enriched at regions carrying repressive histone marks (Izzo et al. 2013). H1 binding might be more sensitive to initiation of transcription than to transcriptional elongation (Izzo et al. 2013). Linker histones may operate in conjunction with a ‘network’ of other chromatin-binding proteins so as to define permissive (euchromatin) and repressive (heterochromatin) DNA domains (Ausio 2006).
H1.4^a^	HIST1H1E	6p22.2	219	
H1.5^a^	HIST1H1B	6p22.1	226	
H1x^b^	H1FX	3q21.3	213	
H1oo	H1FOO	3q22.1	346	
H1t	HIST1H1T	6p22.2	207	
Testis-specific H1	H1FNT	12q13.11	255	
Spermatid-specific H1	HILS1	17q21.33	231	

^a^Gene expressed in a replication-dependent manner

^b^Gene expressed in a replication-independent manner

### Histone H2A

Histone H2A proteins are composed of ~130 amino acid residues, but atypical variants (macroH2As, H2A.X and H2A-Bbd) differ in size. Approximately 265 different members of histone H2A were identified from a variety of species (http://www.actrec.gov.in/histome/). In humans, nineteen variants of histone H2A encoded by 26 genes were reported (Table 2).

**Table 2:** Variants of histone H2A in humans

Variants	Name of genes	Genomic location (Ensembl)	Protein length (aa) (Swiss-Prot)	Function (reference)
H2A type 1	HIST1H2AI	6p22.1	130	Stabilization of the histone core octamer (Ausio 2006)
	HIST1H2AK			
	HIST1H2AL			
	HIST1H2AM			
	HIST1H2AG			
H2A1 type 1-A	HIST1H2AA	6p22.2	131	
H2A type1-B/E	HIST1H2AE	6p22.2	130	
	HIST1H2AB			
H2A type 1-C	HIST1H2AC	6p22.2	130	
H2A type 1-D	HIST1H2AD	6p22.2	130	
H2A type 1-H	HIST1H2AH	6p22.1	128	
H2A type 1-J	HIST1H2A	6p22.1	128	
H2A type 2-A	HIST2H2AA4	1q21.2	130	
	HIST2H2AA4			
H2A type 2-B	HIST2H2AB	1q21.2	130	
H2A type 2-C	HIST2H2AC	1q21.2	129	
H2A type 3	HIST3H2A	1q42.13	130	Unknown function
H2A-Bbd type 1	H2AFB1	Xq28	115	Transcription activation (Tolstorukov et al. 2012)
H2A-Bbd type 2/3	H2AFB2	Xq28	115	Transcription activation (Tolstorukov et al. 2012)
	H2AFB3			
H2A.J	H2AFJ	12p12.3	129	unknown function
H2A.X	H2AFX	11q23.3	143	Genome integrity: DNA repair regulation (Pusarla and Bhargava 2005)
H2A.Z.1	H2AFZ	4q23	128	Maintenance of heterochromatin, transcription repression and activation (Fan et al. 2004; Guillemette et al. 2005; Raisner et al. 2005)
H2A.Z.2	H2AFV	7p13	128	
macroH2A.1	H2AFY	5q31.1	372	Silencing: enriched in inactivated chromosome X (Pusarla and Bhargava 2005)
macroH2A.2	H2AFY2	10q22.1	372	Silencing: enriched in inactive X-chromosome chromatin and in senescence-associated heterochromatin (Pusarla and Bhargava 2005)

### Histone H2B

Except for four variants, the variants of histone H2B contain 126 amino acid residues. The histone H2B family contains 214 different members described from diverse species (http://www.actrec.gov.in/histome/). Histone H2B forms a dimer with histone H2A in nucleosome cores. Histone H2B has 19 variants encoded by 23 genes in humans, the majority of which are assembled in cluster 1 (i.e., 6p22.1–22.2) (Table 3). There are relatively few PTMs identified among the amino acid residues of histone H2B compared to other core histones.

**Table 3:** Variants of histone H2B in humans

Variants	Name of genes	Genomic location (Ensembl)	Protein length (aa) (Swiss-Prot)	Function (references)
H2B type 1-A	HIST1H2BA	6p22.2	127	Specific role of H2B variants is poorly understood. It is probable that they specialize in chromatin compaction and transcription repression, particularly during gametogenesis (Kamakaka and Biggins 2005)
H2B type 1-B	HIST1H2BB	6p22.2	126	
H2B type 1-c/E/F/G/I	HIST1H2BG	6p22.2	126	
	HIST1H2BF			
	HIST1H2BE			
	HIST1H2BI			
	HIST1H2BC			
H2B type 1-D	HIST1H2BD	6p22.2	126	
H2B type 1-H	HIST1H2BH	6p22.2	126	
H2B type 1-J	HIST1H2BJ	6p22.1	126	
H2B type 1-K	HIST1H2BK	6p22.1	126	
H2B type 1-L	HIST1H2BK	6p22.1	126	
H2B type 1-M	HIST1H2BM	6p22.1	126	
H2B type 1-N	HIST1H2BN	6p22.1	126	
H2B type 1-O	HIST1H2BN	6p22.1	126	
H2B type 2-E	HIST2H2BE	1q21.2	126	
H2B type 2-F	HIST2H2BF	1q21.2	126	
H2B type 3-B	HIST3H2BB	1q42.13	126	
H2B type F-M	H2BFM	Xq22.2	257	
H2B type F-S	H2BFS	21q22.3	126	
H2B type W-T	H2BFWT	Xq22.2	175	
putative H2B type 2-C	HIST2H2BC	1q21.2	193	
putative H2B type 2-D	HIST2H2BD	1q21.2	164	

### Histone H3

Histone H3 consists of ~136 amino acid residues; only the centromere protein A (CENP-A) is a longer variant. The histone H3 family contains 216 different members characterized from various species (http://www.actrec.gov.in/histome/). In humans, 20 genes encode 8 variants of histone H3, most of which are clustered on chromosome 6 (Table 4). Histone H3 is the most extensively post-translationally modified of the five histones.

**Table 4:** Variants of histone H3 in humans

Variants	Name of genes	Genomic location (Ensembl)	Protein length (aa) (Swiss-Prot)	Function (references)
H3.1^a^	HIST1H3A	6p22.2	136	Replication and DNA repair (Szenker et al. 2011)
	HIST1HAD			
	HIST1H3C			
	HIST1H3E			
	HIST1H3I			
	HIST1H3G			
	HIST1HAJ			
	HIST1H3H			
	HIST1H3B			
	HIST1H3F			
H3.1t	HIST3H3	1p42.3	136	Chromatin reorganization during spermatogenesis (Witt et al. 1996)
H3.2^a^	HIST2H3C	1q21.2	136	Replication and DNA repair (Szenker et al. 2011)
	HIST2H3A			
	HIST2H3D			
H3.3^b^	H3F3A	1p42.2	136	Transcription activation (Mito et al. 2005)
	H3F3B	17q25.1		
H3.3C (also named H3.5)	H3F3C	12p11.21	135	Transcription activation (Schenk et al. 2011)
H3.X^b^	H3X	5p15.1		Protein not detected in vivo (Wiedemann et al. 2010)
H3.Y^b^	H3Y	5p15.1		Probable regulation of cellular responses to stress, transcription activation (Wiedemann et al. 2010)
H3-like centromeric protein A (CENP-A)	CENPA	2p23.3	140	Proper chromosome segregation (Fukagawa 2004)

^a^Gene expressed in a replication-dependent manner

^b^Gene expressed in a replication-independent manner

### Histone H4

Histone H4 contains only 103 amino acid residues and forms a heterotetramer (H3–H4)_2_ with histone H3. The histone H4 family consists of 116 members reported from different organisms (http://www.actrec.gov.in/histome/). Interestingly, humans have a single histone H4 protein encoded by 14 genes, eleven of which are clustered on chromosome 6 (Table 5).

**Table 5:** Histone H4 in humans

Histone	Name of genes	Genomic location (Ensembl)	Protein length (aa) (Swiss-Prot)	Function (references)
H4	HIST4H4	12p12.3	103	No variants are recognized
	HIST1H4A	6p22.1		
	HIST1H4B	6p22.2		
	HIST1H4C	6p22.2		
	HIST1H4D	6p22.2		
	HIST1H4E	6p22.2		
	HIST1H4F	6p22.2		
	HIST1H4H	6p22.2		H4 makes contact with the other three histones in the octamer
	HIST1H4I	6p22.1		
	HIST1H4J	6p22.1		
	HIST1H4K	6p22.1		
	HIST1H4L	6p22.1		
	HIST2H4A	1q21.2		
	HIST2H4B	1p21.2		

## Conclusions

DNA methylation is considered to be a relatively stable epigenetic modification. Recent genome-wide analyses of the DNA methylation in mammalian cells suggest that some enzymes are capable of erasing or modifying existing methylation patterns. Although DNA cytosine methylation is well-characterized, little is known about the role of cytosine derivatives in gene expression regulation. In the future, high-resolution sequencing technologies should enable creation of quantitative maps of 5hmC, 5fC, and 5caC in different cell types. Understanding the dynamics of these modifications can help to explain their role in physiological or pathological conditions. Interestingly, due to subtle sequence divergences, incorporation of histone variants may influence the stability of nucleosome and change the potential of specific histone modifications. Histone variant composition is a key player in shaping chromatin structure; this also should be considered as one of the epigenetic regulation elements. It is well known that epigenetic disturbance may lead to different phenotypes and monogenic or complex diseases as well as oncogenic transformation. We strongly believe that rapidly growing understanding of epigenetic phenomena could bring a breakthrough in the diagnosis and treatment of many disorders. Moreover, better knowledge about the epigenetic etiology of the diseases provides an opportunity to develop innovative new epigenetic drugs.
